# Restless Legs Syndrome in Parkinson’s Disease

**DOI:** 10.3390/jpm13060915

**Published:** 2023-05-30

**Authors:** Ştefania Diaconu, Laura Irincu, Larisa Ungureanu, Bogdan Ciopleiaș, Diana Țînț, Cristian Falup-Pecurariu

**Affiliations:** 1 Department of Neurology, County Clinic Hospital, 500365 Braşov, Romania; valentina.irincu@yahoo.com (L.I.); ungureanuelenalarisa@gmail.com (L.U.); bciopleias@gmail.com (B.C.); crisfp100@yahoo.co.uk (C.F.-P.); 2 Faculty of Medicine, Transilvania University, 500036 Braşov, Romania; dianatint@gmail.com; 3 Clinicco, 500059 Braşov, Romania

**Keywords:** restless legs syndrome, Parkinson’s disease, sleep quality, non-motor symptoms, pain, fatigue

## Abstract

Background: Restless legs syndrome (RLS) might worsen sleep quality and quality of life in people with Parkinson’s disease (PwPD). Objective: The main aim of the present study is to explore the associations between RLS and sleep, quality of life and other non-motor symptoms (NMS) in a sample of PwPD. Methods: We compared the clinical features of 131 PwPD with and without RLS, in a cross-sectional study. We used several validated scales for assessment: the International Restless Legs Syndrome Study Group rating scale (IRLS), Parkinson’s Disease Sleep Scale version 2 (PDSS-2), Parkinson’s Disease Questionnaire (PDQ-39), Non-Motor Symptoms Questionnaire (NMSQ) and International Parkinson and Movement Disorder Society Non-Motor Rating Scale (MDS-NMS). Results: Thirty-five patients (26.71%) out of the total PwPD met the RLS diagnostic criteria, without significant differences between male (57.14%) and female (42.87%) (*p* = 0.431). Higher total scores of PDSS-2 were recorded among PwPD + RLS (*p* < 0.001), suggesting worse sleep quality. Significant correlations were observed between the diagnosis of RLS and some types of pain (especially nocturnal pain), physical fatigue and probable sleep-disordered breathing, according to the MDS-NMSS assessment. Conclusions: RLS has a high frequency in PwPD and it requires proper management, considering its consequences on sleep and quality of life.

## 1. Introduction

Restless legs syndrome (RLS) is defined by disturbing symptoms in lower limbs, occurring in periods of inactivity (especially at night), that lead to the need for movement. Various types of movements may partially or totally relieve these abnormal feelings [1]. In 2014, the International RLS Study Group (IRLSSG) established five mandatory criteria for the diagnosis of RLS; in addition to the aforementioned characteristics, these unpleasant symptoms should not be the result of other medical or behavioral conditions [2]. RLS can occur at any age throughout the patient’s lifetime [3] and is often known to have a gradual evolution, sometimes with an intermittent course or with complete remission, especially in secondary forms of RLS [1].

In the general population, a high prevalence of RLS was reported (up to 10%) [4], RLS being more commonly encountered in females [5]. Secondary causes of RLS have also been identified: renal failure [6], hepatic cirrhosis [7] and pregnancy (generally with a tendency to decrease after delivery) [8]. On the other hand, RLS can be encountered in other less-common conditions, such as rheumatoid arthritis [9], fibromyalgia [10], multiple sclerosis [11] or systemic lupus erythematosus [12].

In Parkinson’s disease (PD), the mean prevalence of RLS was 15.74% [13]. RLS is a common condition, similar with other underreported motor or non-motor symptoms, such as hiccups, hallucinations [14], impulse control disorders or sexual dysfunctions [15]. In case-control studies, the mean prevalence of RLS was higher in people with PD (12%) vs. healthy controls (5.1%) [13]. In PwPD, diagnosis of RLS is challenging due to multiple confounders (motor fluctuations, akathisia, neuropathy) [16]. One four-year longitudinal study reported an increase in RLS prevalence from 4.6% to 16.3% along with disease progression [17]. There are several theories suggesting a possible link between dopaminergic dysfunction in PD and RLS, primarily based on the effectiveness of dopaminergic treatment in alleviating the symptoms of RLS [18]. Other possible common mechanisms may include abnormal iron metabolism or the involvement of the adrenergic system. On the contrary, RLS and PD may have completely different pathophysiology or may be in fact different entities; therefore, further research is necessary to investigate the link between these two conditions, taking into account the impact of RLS on quality of life [18].

In the general population, RLS commonly leads to sleep disturbances and may have a negative impact on quality of life, though it represents an underdiagnosed syndrome [19]. In PwPD, RLS has been reported to impair sleep parameters and quality of sleep [20,21]. Previous studies have also demonstrated an association between RLS and anxiety and depression [22,23]. Considering the important link between RLS and impaired sleep, a personalized approach to the patient with PD and sleep disturbances (including insomnia) should also include the investigation of the pre-sleep habits and symptoms, and therefore the existence of RLS symptoms [24].

Few studies explored the correlations between RLS and other non-motor symptoms (NMS) in PwPD.

The main aims of this study were: to explore the frequency and the characteristics of RLS in a sample population of PwPD; to establish the correlations between RLS and other related sleep disturbances and the impact of RLS on sleep quality in PwPD; and to identify the correlations between RLS and other non-motor symptoms.

## 2. Materials and Methods

### 2.1. Patients and Study Design

A questionnaire-based cross-sectional study on 131 people with PD (PwPD) was conducted at the Department of Neurology of the County Clinical Hospital, Transilvania University of Braşov, Romania. Inclusion criteria were: diagnosis of PD (based on MDS clinical diagnosis criteria [25]), regardless of the severity of the disease; absence of severe cognitive impairments, according to the Mini Mental State Examination Test; and willingness to participate in the study. Exclusion criteria were: secondary parkinsonism; atypical forms of parkinsonism; and comorbidities known to cause secondary RLS (iron deficiency and renal or hepatic diseases).

Clinical assessments included: demographic information (age, gender, age at PD onset), levodopa equivalent daily dose (LEDD), duration of the disease, Hoehn & Yahr (H&Y) stages in both “On” and “Off” states, comorbidities and medication for sleep or for other associated medical conditions. Patients were evaluated by a specialist in neurology with a battery of validated scales for assessing RLS, sleep, cognition and motor function, which will be further described. The Movement Disorder Society Unified Parkinson’s Disease Rating Scale (MDS-UPDRS)—part III and the SCales for Outcomes in PArkinson’s disease (SCOPA)-Motor evaluation was performed during “On” states. All patients included in this study signed informed consent forms. The protocol for the present study was admitted by the Ethics Committee of University Transilvania of Braşov (1.11/01/2019), and the study was conducted in compliance with the Declaration of Helsinki.

### 2.2. Questionnaires

Regarding the diagnosis of RLS, all participants were initially assessed using the RLS diagnostic criteria; patients fulfilling all the mandatory criteria were considered as PwPD + RLS and were further evaluated with the International Restless Legs Syndrome Study Group rating scale (IRLS) [26], which consists of 10 items regarding the RLS characteristics, the presence of sleep disturbances, symptom frequency, overall and daily severity, impact on daily activities and the effects on mood. For each question, possible answers ranged from 4 (very severe) to 0 (none). The total IRLS score was used for establishing the severity of RLS: very severe (>31), severe (21–30), moderate (11–20), and mild (0–10) [26]. Sleep characteristics were assessed with several validated scales. Parkinson Disease Sleep Scale (PDSS)-2 [27] is the revised version of PDSS-1 [28]; it is specifically designed to be applied to the PD population and it consists of 15 items regarding various sleep issues. Each item is scored from 0 (never) to 4 (very frequent), and higher total values indicate more severe sleep problems [27]. In order to evaluate insomnia, we used the Insomnia Severity Index (ISI) [29], which is a brief tool containing 7 questions designed to evaluate the severity of insomnia and its consequences. Daytime sleepiness was evaluated with the Epworth Sleepiness Scale (ESS) [30], one of the most used scales for this scope. It indicates the possibility of falling asleep in eight common active or passive situations. The Parkinson’s Disease Questionnaire (PDQ-39) [31] is a widely use tool consisting of 39 questions, designed to investigate the impact of PD in eight health domains. The Non-Motor Symptoms Questionnaire (NMSQ) [32] is a brief, validated screening questionnaire which is used in clinical practice to easily detect the presence of non-motor symptoms (NMS) in the previous month. For all the 30 questions representing various NMS, the patient answers with ‘yes’ or ‘no’, the maximum score being 30. The Non-Motor Symptoms Scale (NMSS) was designed to improve the information obtained with the NMSQ; therefore, the severity and the frequency of the symptoms were added [33]. Furthermore, a revised version of the NMSS was published recently: the International Parkinson and Movement Disorder Society Non-Motor Rating Scale (MDS-NMSS). This scale assesses the frequency and severity of more domains and symptoms than NMSS, and it also contains a scale for the evaluation of non-motor fluctuations [34]. We evaluated depression and anxiety using the Hospital Anxiety and Depression Scale (HADS), which is composed of two scores: one for depression and one for anxiety, with a maximum score of 21 for each score (a higher score suggesting worse symptoms) [35]. Cognition was evaluated with the Mini-Mental State Examination (MMSE) and Montreal Cognitive Assessment (MoCA) scales, which proved their utilization in clinical practice, including on patients with neurodegenerative disorders [36,37]. Severity of the disease (regarding the motor functioning) was assessed using the following scales: H&Y Staging, MDS-UPDRS parts III and IV, and SCOPA-Motor [38].

### 2.3. Data Analysis

Statistical analyses were performed with IBM SPSS Statistics for Windows, Version 23.0. Armonk, NY: IBM Corp. Probability values of *p* < 0.05 were considered significant. Descriptive data were shown as means +/− standard deviations or percentages. An asymmetric sample distribution was determined using the Shapiro–Wilk test. Fisher’s exact test and Mann–Whitney test were used to compare the characteristics of PwPD + RLS and PwPD − RLS. A Kruskal–Wallis test was used to compare subscale means across groups.

## 3. Results

We included in the study 131 consecutive PD patients. Demographic aspects are presented in Table 1.

A total of 35 patients (26.71%) fulfilled the IRLSSG criteria for RLS. The mean IRLS values were 14.74 ± 6.103, while, according to the severity of IRLS, 7 patients presented mild intensity of their complaints, 21 presented moderate severity, and 7 presented severe complaints. None of our patients reported very severe symptoms.

There were no significant differences between groups (PwPD + RLS vs. PwPD − RLS) regarding age, gender, age at onset of PD, duration of PD and LEDD. The mean age of the PwPD + RLS sample was 74.51 (±8.38) years, and the mean age in the PwPD − RLS group was 74.38 (±9.23) years. In our sample, 15 (42.85%) of the subjects were female in the PwPD + RLS group, and 50 (52.08%) in the PwPD − RLS group. The mean PD duration was 6.43 (±5.37) years in the PwPD + RLS group, and 4.58 (±3.66) in the PwPD − RLS group, respectively. No significant differences were found regarding LEDD between groups (459.88 mg in PwPD + RLS and 505.4 mg in PwPD − RLS, respectively) (Table 1). Moreover, no significant differences were noted concerning the doses of levodopa or dopamine agonists between the two groups.

Regarding the motor features, we found significant differences in the MDS-UPDRS III total scores between the two populations (36.45 ± 11.02 in the PwPD + RLS group and 31.17 ± 14.22 in the PwPD − RLS group, respectively; *p* = 0.009), but no other significant differences in H&Y staging, MDS-UPDRS IV scores and SCOPA-Motor total scores (Table 2).

**Figure 1:**
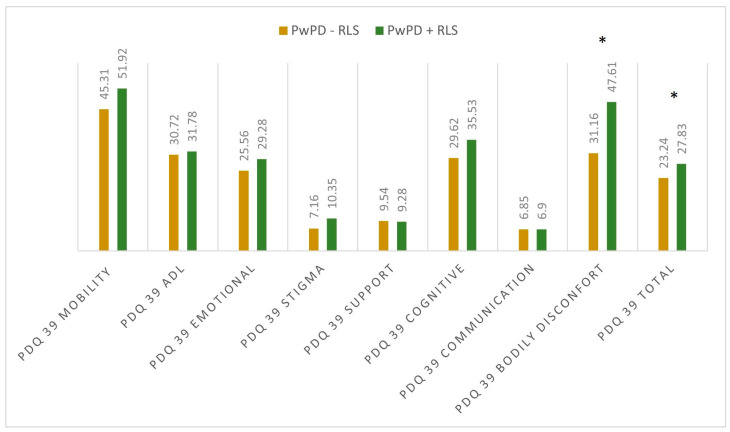
Mean scores in the dimensions of PDQ 39 between the two groups. Significant differences were marked with asterisk (*), and were seen in the dimension bodily discomfort (*p* < 0.001) and PDQ 39 total score (*p* = 0.046); Mann–Whitney U Test. PD: Parkinson’s disease; PDQ-39: Parkinson’s Disease Questionnaire; PwPD: people with Parkinson’s disease; RLS: restless legs syndrome.

**Figure 2:**
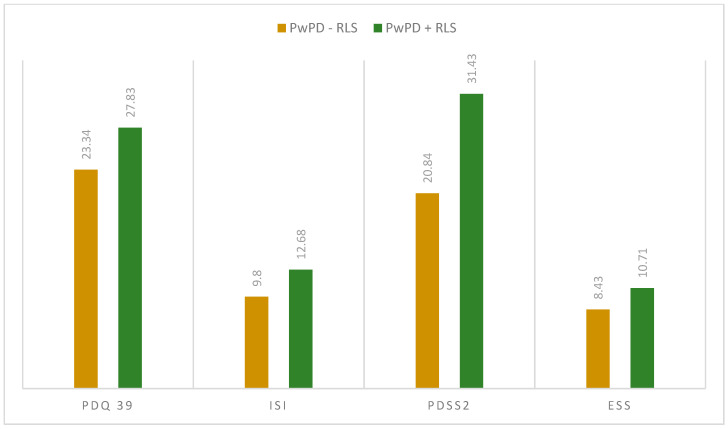
Mean scores in the applied scaled between the two groups (PwPD + RLS vs. PwPD − RLS); Mann–Whitney U Test. ESS: Epworth Sleepiness Scale; ISI: Insomnia Severity Index; PD: Parkinson’s disease; PDQ-39: Parkinson’s Disease Questionnaire; PDSS-2: Parkinson Disease Sleep Scale 2; PwPD: people with Parkinson’s disease; RLS: restless legs syndrome.

When assessing the sleep quality and sleep disturbances, we observed differences regarding PDQ-39 total scores (27.83 ± 12.54 in PwPD + RLS; 23.34 ± 15.09 in PwPD − RLS; *p* = 0.046) and PDSS-2 total scores (31.43 ± 10.18 in PwPD + RLS; 20.84 ± 9.91 in PwPD − RLS; *p* < 0.001), as shown in Figure 1 and Figure 2.

**Figure 3:**
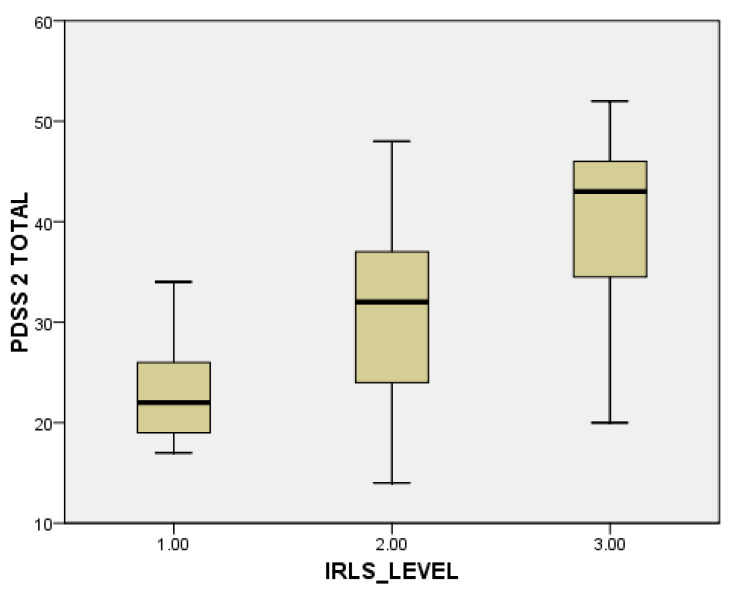
Severity of PDSS-2 scores, in accordance with the severity of the IRLS scale (1 = mild; 2 = moderate; 3 = severe). IRLS: International Restless Legs Syndrome Study Group rating scale; PDSS-2: Parkinson Disease Sleep Scale-2.

When evaluating individual domains of PDQ-39, significant differences were seen in the dimension bodily discomfort (*p* < 0.001) in PwPD + RLS (Figure 1). PwPD also presented higher rates of insomnia, compared to individuals without RLS (ISI total scores of 12.68 ± 5.98 in PwPD + RLS and 9.8 ± 5.91 in PwPD − RLS, *p* = 0.016)**.** Excessive daytime sleepiness was more prominent in the PwPD + RLS group, according to higher ESS scores (10.71 ± 4.91 in PwPD + RLS, 8.43 ± 5.61, *p* = 0.02), as shown in Table 2, Figure 2. Moreover, depression and anxiety were observed more commonly in the PwPD + RLS group compared with the PwPD − RLS group, according to the HADS depression and anxiety scores (Table 2). No differences were observed regarding the MMSE and MOCA scores in both groups. We also assessed whether the severity of RLS symptoms has any consequences on sleep and if it is correlated with the severity of the RLS. In our study group, we found that total scores of PDSS-2 were higher according to the IRLSS severity (Figure 3), showing a worse quality of sleep in patients with worse RLS symptoms.

When applying the NMSQ to evaluate the NMS, we found significant correlations only regarding question 26, related to the unpleasant sensations in legs at night (*p* < 0.001). Moreover, several other domains of the NMS spectrum were observed when analysing the MDS-NMS. We found significant higher scores in PwPD + RLS compared with PwPD − RLS regarding the following symptoms and domains: restlessness, periodic limb movements, snoring/gasping/difficulty breathing, MDS-NMS ‘K. Sleep and wakefulness’ domain total score, other types of pain (nocturnal, orofacial), MDS-NMS ‘L. Pain’ domain total score, physical fatigue and MDS-NMS total score (Table 3).

**Figure 4:**
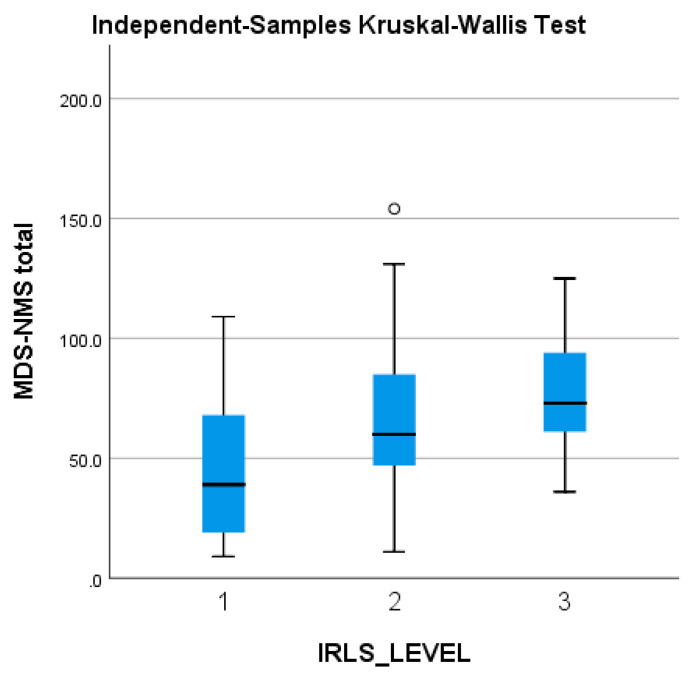
Severity of the MDS-NMS total score, in accordance with the severity of the IRLS scale (1 = mild; 2 = moderate; 3 = severe). IRLS: International Restless Legs Syndrome Study Group rating scale; MDS-NMSS: International Parkinson and Movement Disorder Society Non-Motor Rating Scale; PD: Parkinson’s disease. The ° represents an outlier from the median score.

The MDS-NMS total score tends to be higher in patients with worse RLS symptoms (and higher IRLS scores), but without reaching statistical significance (Figure 4).

## 4. Discussion

The prevalence of RLS symptoms in the study group was 26.71%, in line with the previous reported prevalences. Swensson et al. reported a prevalence of 24% in a sample of 113 PwPD [39]. One cross-sectional study including more than 300 PwPD reported a prevalence of 20.8% [40]. In another study, out of 114 PwPD patients, 21.9% individuals were diagnosed with RLS [41]. The variability of RLS prevalence may be explained due to various criteria used for diagnosis; moreover, several related symptoms, such as motor fluctuations or polyneuropathy, may be confounders in RLS diagnosis.

In our study sample, there were no significant differences regarding gender, the age of PD onset, and PD duration between PwPD + RLS and PwPD − RLS. However, there was a significant correlation of RLS positive diagnosis and motor severity, as evaluated with the MDS-UPDRS-III score (*p* = 0.009). Similar to our results, most studies did not find any relevant association between RLS and gender [42,43,44]. According to some research [45,46], RLS was more commonly encountered in females, with possible connections to hormonal factors or iron deficiency. The association between RLS and age is controversial; Ondo et al. [40] did not observe any differences between the ages of patients with RLS vs. patients without RLS. On the contrary, Krishan et al. [47] reported more RLS symptoms in older patients, while Nomura et al. [48] identified RLS in younger people. Previous studies have not found significant differences in the severity of PD assessed by H&Y stages between patients with and without the diagnosis of RLS [20,46,47], but this aspect is under controversy as well. Swensson et al. observed a higher frequency of RLS symptoms in stages 1 and 2 H&Y compared with stages 3 and 4, but patients with advanced PD (H&Y 5) were not included in the study [39]. Similarly to our findings, Piao et al. [49] and Shin et al. [50] observed that PwPD and diagnosis of RLS have higher scores on motor assessments. Patients with more advanced stages of PD being more likely to develop RLS could suggest the involvement of the neurodegeneration process in both PD and RLS. However, transcranial brain sonography showed no differences in echogenicity of substantia nigra between PwPD with or without RLS, while idiopathic RLS was characterized by significant hypoechogenicity, suggesting different pathogenic mechanisms that might explain idiopathic RLS and RLS in PwPD [51]. Moreover, one SPECT (Single-photon emission computed tomography) study revealed no similar patterns between individuals with RLS and those with early PD [52]. Involvement of other anatomical structures than the nigrostriatal pathway is plausible (such as the diencephalic-spinal tract, as shown in animal models [53]).

In our study group, higher total PDSS-2 scores (suggesting worse sleep quality) were identified in PwPD + RLS patients (mean score 31.43 ± 10.18) as compared with those without RLS (mean score 20.84 ± 9.91, *p* < 0.001). Moreover, the severity of PDSS-2 correlates with the severity of the RLS symptoms, as measured with IRLS. The significant association of RLS with sleep disorders was also observed in previous studies, although the first version of PDSS was used. PwPD + RLS reported lower scores of PDSS-1 than in PwPD - RLS, suggesting higher severity of the sleep problems in the presence of RLS [20,41,54]. To the best of our knowledge, there were no previous studies evaluating sleep symptoms in RLS using the PDSS-2 version.

Our findings suggest that PwPD + RLS have a worse sleep quality and a reduced quality of life compared with PwPD − RLS, as evaluated with the PDQ-39 and PDSS-2 scales. When evaluated with the PDQ-39 score, the most significant differences were noticed in the bodily discomfort dimension (*p* < 0.001) and PDQ-39 total score (*p* = 0.046), the worse scores being observed in the PwPD + RLS group. One study including 187 patients with parkinsonian syndromes showed that the frequency of RLS is highest in the PD group and is associated with reduced sleep quality and daytime sleepiness [20]. In another study, in which 114 PwPD were evaluated, no significant differences were noticed between the total scores of PDQ-39 in PwPD + RLS compared to PwPD − RLS; however, when individual components of PDQ-39 were assessed, the bodily discomfort dimension was rated the highest by PwPD + RLS [41].

We observed an association between RLS and excessive daytime sleepiness, as evaluated with ESS. In line with our results, several other studies have demonstrated an association between RLS and daytime sleepiness [20,44,55,56].

No differences were observed regarding cognitive function in our study group, independent of the positive RLS diagnosis. Similarly, You et al. have not identified an association between RLS and cognitive dysfunction [54]. Cognitive decline is commonly observed in advanced stages of PD and may be related to acetylcholine dysfunction rather than dopamine, which is known to have more implications in RLS [49].

Other NMS may also be associated with RLS. One study including 74 PwPD reported more NMS such as anxiety, depression and dysautonomia in patients who were also diagnosed with RLS [54]. The aim of the study conducted by Rana et al. [23] was to determine if RLS can be a risk factor for anxiety and depression in PwPD. Therefore, in the study were included 27 PwPD + RLS, 27 PwPD − RLS and 27 healthy controls. The lowest scores for anxiety and depression were reported in the control group, but no significant differences were observed in PwPD, independently of the diagnosis of RLS. Another case-control study demonstrated that RLS represents an independent risk factor for anxiety in PwPD [22]. We also found significant correlations between the diagnosis of RLS and anxiety and depression, as evaluated with HADS. Low concentrations of dopamine and 5-hydroxytriptamine in the cerebral spinal fluid of PwPD + RLS compared to PwPD − RLS may explain the common association of RLS with depression and anxiety [49].

To the best of our knowledge, this is the first study to explore the profile of NMS in people diagnosed with both PD and RLS. For this purpose, we used the NMSQ and the newly developed MDS-NMS. Our findings reveal a correlation between the diagnosis of RLS and the following NMS/domains in PwPD + RLS vs. PwPD − RLS: restlessness, periodic limb movements, snoring/gasping/difficulty breathing, MDS-NMS ‘K. Sleep and wakefulness’ domain total score, other types of pain (nocturnal, orofacial), MDS-NMS ‘L. Pain’ domain total score, physical fatigue and MDS-NMS total score. The 26th question of the NMSQ and the 4th and 5th items of the subscale ‘K. Sleep and Wakefulness’ of the MDS-NMSS are suggestive for symptoms characterising RLS, though the use of the five criteria described for the diagnosis of RLS is advisable. Even if we found significant correlations between RLS and other validated scales for the assessment of insomnia and EDS, we did not find correlations with those corresponding items of NMSQ or MDS-NMSS. We observed an association between RLS and total score of the MDS-NMS ‘K. Sleep domain total score’, though. Also, the burden of NMS is broader as the severity of RLS increases, but this finding was not statistically significant.

All the 74 patients with idiopathic RLS included in the study conducted by Sauerbier et al. reported at least two NMS, the most-reported being insomnia, followed by nocturia and sad feelings [57]. In one longitudinal study including 109 PwPD assessed also with NMSQ, the non-motor symptoms that were prone to be correlated with RLS were insomnia (at RLS diagnosis), dizziness and daytime sleepiness (at follow-up), in comparison with PwPD − RLS [17]. The presence of dysautonomia in PwPD + RLS, especially of the cardiovascular system, was also demonstrated by You et al., using the SCOPA-AUT scale [54]. Sobreira-Neto at al. found an association between insomnia, olfactory dysfunction, and constipation in PwPD + RLS, but the assessments for the olfaction and constipation were done based on a clinical interview and not by standardized validated tools [58]. 

In our study group, we observed an association between RLS and item 4 of the ‘L. Pain’ domain of the MDS-NMS, and with the total score of this domain. We suppose that patients with RLS reported more frequent and severe pain, especially nocturnal pain, due to the unpleasant symptoms in the lower limbs that may be painful for some of them. Similarly to our results, previous studies have shown that there is a link between pain and RLS (PwPD reporting pain reported more severe RLS symptoms [59], and also PwPD + RLS reported more pain than PwPD − RLS and patients with RLS but no PD or healthy controls [60]).

We found a correlation between the presence of the RLS diagnosis and physical fatigue. Associations between fatigue and RLS in PwPD have been scarcely described in previous studies. Ylikosi et al. have demonstrated in a questionnaire-based study that fatigue, among other sleep disturbances, was more common in PwPD + RLS [61]. The questionnaire used in that study included one question related to the presence of fatigue during daytime [61]. Using the Fatigue Severity Scale, Fereshtehnejad et al. showed the presence of fatigue in PwPD + RLS patients, but the results did not reach statistical significance [42].

Moreover, our results show a correlation between RLS and the probability of OSA. Item 6 of the domain K. ‘Sleep and Wakefulness’ of the MDS-NMS asks if the patient has woken at night due to snoring, gasping, or difficulty breathing. In the general population, there have been studies demonstrating an association between RLS and OSA [62,63]; the causal relationships between these two sleep disorders are still unclear [64].

We acknowledge that our study has several limitations, including: small sample size (there were only 35 patients in the RLS group); the absence of objective measures of the sleep parameters, which would be useful to confirm our findings obtained from questionnaires; the absence of a control group for proper comparisons; and the lack of paraclinical investigations such as serum iron or ferritin levels, which would be helpful to exclude other secondary causes of RLS. We did not assess the temporal relation between the onset of RLS and the diagnosis of PD, which would be informative for the bidirectional/potential common causative links between RLS and PD. Other possibly associated sleep disturbances such as sleep apnoea, periodic limb movements, REM sleep behaviour or OSA were not evaluated separately. We observed associations between RLS and other types of pain (nocturnal pain) and physical fatigue according to the corresponding items of the NMSQ/MDS-NMSS, but we did not assess these correlations with other specifically designed tools for the assessment of pain and fatigue. Patients were examined in the “On” stage; therefore, the influence of medication on sleep or RLS was not explored.

## 5. Conclusions

RLS is commonly encountered in PwPD and is related to impaired quality of sleep, reduced quality of life and other non-motor symptoms, such as pain, fatigue and breathing difficulties during sleep. Proper investigation and management of this symptom should be considered in order to improve the quality of life and the quality of sleep in patients diagnosed with PD.

## Figures and Tables

**Table 1:** Demographic data in PwPD + RLS vs. PwPD − RLS (Fisher’s exact test).

	PwPD + RLS N = 35 (M/F)	PwPD − RLS N = 96 (M/F)	*p*-Value
Sex	20/15	46/50	0.431
LEDD (mean, mg)	459.88	505.4	0.434
Age (years ± SD)	74.51 (±8.38)	74.38 (±9.23)	0.325
Age at PD onset (years ± SD)	68.303 (±10.69)	69.66 (±9.89)	0.347
Duration of PD (years ± SD)	6.43(±5.377)	4.58 (±3.66)	0.367

LEDD: Levodopa equivalent daily dose; PD: Parkinson’s disease; PwPD: people with Parkinson’s disease; RLS: restless legs syndrome; SD: standard deviation.

**Table 2:** Main characteristics regarding motor and non-motor symptoms between the two groups (PwPD + RLS vs. PwPD − RLS; Mann–Whitney U Test).

	PwPD + RLS N = 35	PwPD − RLS N = 96	*p*-Value
Motor symptoms			
MDS-UPDRS III	36.45 (±11.02)	31.17 (±14.22)	**0.009**
MDS-UPDRS IV	3.46 (±2.61)	3.44 (±3.27)	0.114
H&Y	2.42 (±0.60)	2.38 (±0.79)	0.415
SCOPA-Motor	24.97 (±7.19)	21.61 (±9.34)	0.009
Non-motor symptoms			
IRLSSG	14.74 (±6.10)	–	<0.001
PDQ 39	27.83 (±12.54)	23.34 (±15.09)	**0.046**
ISI	12.68 (±5.98)	9.8 (±5.91)	**0.016**
PDSS 2	31.43 (±10.18)	20.84 (±9.91)	**<0.001**
ESS	10.71 (±4.91)	8.43 (±5.61)	**0.02**
MMSE	27.54 (±2.33)	27.19 (±3.69)	0.786
MoCA	23.71 (±4.7)	23.33 (±5.8)	0.938
HADS Depression	16.91 (±6.89)	13.40 (±7.85)	**0.013**
HADS Anxiety	6.55 (±3.71)	4.97 (±3.76)	**0.027**
HADS Total	10.35 (±3.61)	8.43 (±4.50)	**0.016**

ESS: Epworth Sleepiness Scale; H&Y: Hoehn & Yahr; HADS: Hospital Anxiety and Depression Scale; IRLSSG: International Restless Legs Syndrome Study Group rating scale; ISI: Insomnia Severity Index; MMSE: Mini-Mental State Examination; MoCA: Montreal Cognitive Assessment; PD: Parkinson’s disease; PDQ-39: Parkinson’s Disease Questionnaire; PDSS-2: Parkinson Disease Sleep Scale - 2; PwPD: people with Parkinson’s disease; RLS: restless legs syndrome; SCOPA: Scales for Outcomes in Parkinson’s disease; MDS-UPDRS: Movement Disorders Society Unified Parkinson’s Disease Rating Scale. Bold values denote statistical significance (*p* < 0.05).

**Table 3:** The scores of specific items of MDS-NMS in the two groups (PwPD + RLS vs. PwPD − RLS), Mann–Whitney Test.

	PwPD + RLS		PwPD − RLS		
	Mean	Std. Deviation	Mean	Std. Deviation	*p*
MDS-NMS A. Depression domain total score	3.629	3.5570	3.629	3.5570	0.058
MDS-NMS B. Anxiety total score	4.000	5.5572	4.000	5.5572	0.126
MDS-NMS C. Apathy total score	1.886	2.0113	1.886	2.0113	0.183
MDS-NMS D. Psychosis total score	0.314	0.8668	0.314	0.8668	0.953
MDS-NMS E. Impulse Control Disorders total score	0.029	0.1690	0.029	0.1690	0.463
MDS-NMS F. Cognition total score	6.514	8.1615	6.514	8.1615	0.850
MDS-NMS G. Orthostatic hypotension total score	3.229	6.2830	3.229	6.2830	0.703
MDS-NMS H. Urinary total score	7.029	6.2755	7.029	6.2755	0.314
MDS-NMS I. Sexual total score	0.314	1.5295	0.314	1.5295	0.191
MDS-NMS J. Gastrointestinal total score	2.000	2.4254	2.000	2.4254	0.941
MDS-NMS K. Sleep total score	15.229	10.3216	15.229	10.3216	**<0.001**
Restlessness fr x sev	3.057	3.0673	3.057	3.0673	**<0.001**
Periodic limb movements fr x sev	1.343	2.1955	1.343	2.1955	**<0.001**
Snoring, difficulty breathing fr x sev	2.000	2.1693	2.000	2.1693	**<0.001**
MDS-NMS L. Pain total	11.743	8.4029	11.743	8.4029	**0.003**
Other types of pain (nocturnal, orofacial) fr x sev	3.714	3.6346	3.714	3.6346	**0.002**
MDS-NMS M. Other non-motor symptoms total	11.086	7.9164	11.086	7.9164	0.177
Physical fatigue fr x sev	5.771	4.2084	5.771	4.2084	**0.007**
MDS-NMS total	67.000	35.4119	67.000	35.4119	**0.007**

fr: frequency; MDS-NMSS: International Parkinson and Movement Disorder Society Non-Motor Rating Scale; PD: Parkinson’s disease; PwPD: people with Parkinson’s disease; RLS: restless legs syndrome; sev: severity. Bold values denote statistical significance (*p* < 0.05).

## Data Availability

The data presented in this study are available on reasonable request from the corresponding author.
